# Photophobia in migraine: A symptom cluster?

**DOI:** 10.1177/03331024211014633

**Published:** 2021-05-14

**Authors:** Arnold J Wilkins, Sarah M Haigh, Omar A Mahroo, Gordon T Plant

**Affiliations:** 1 Department of Psychology, University of Essex, UK; 2 Department of Psychology and Integrative Neuroscience, University of Nevada, Reno, USA; 3 Institute of Ophthalmology, University College London, London, UK and Retinal Service, Moorfields Eye Hospital, London, UK; 4 University College London, London, UK

**Keywords:** photophobia, migraine, pattern glare, flicker, colour contrast

## Abstract

Photophobia is one of the most common symptoms in migraine, and the underlying mechanism is uncertain. The discovery of the intrinsically-photosensitive retinal ganglion cells which signal the intensity of light on the retina has led to discussion of their role in the pathogenesis of photophobia. In the current review, we discuss the relationship between pain and discomfort leading to light aversion (traditional photophobia) and discomfort from flicker, patterns, and colour that are also common in migraine and cannot be explained solely by the activity of intrinsically-photosensitive retinal ganglion cells. We argue that, at least in migraine, a cortical mechanism provides a parsimonious explanation for discomfort from all forms of visual stimulation, and that the traditional definition of photophobia as pain in response to light may be too restrictive. Future investigation that directly compares the retinal and cortical contributions to photophobia in migraine with that in other conditions may offer better specificity in identifying biomarkers and possible mechanisms to target for treatment.

## Introduction

Photophobia occurs in a wide range of ophthalmic, neurological and behavioural conditions, the commonest of which is migraine. This review is restricted to the photophobia that occurs in migraine. The literal meaning of photophobia is fear of light (1), but this is an oversimplification of the experience of migraine sufferers. In migraine, both headache and behavioural evidence of aversion can be provoked in response to four categories of retinal stimulation: bright light (2), flickering light (even when the flicker is too rapid to be seen [3]), patterns (4–6) and colour (7–9). The mechanisms may differ during and between acute attacks where headache is manifest. Our aim therefore in this review is to suggest a mechanism for *interictal* migraine photophobia that encompasses all four categories of visual stimulation and of aversion to light other than headache: thereby we argue for a broadening of the concept of photophobia in migraine. We review the physiological mechanisms underlying the various types of photophobia – that from bright light, flicker, patterns, and colour - and provide a parsimonious explanation.

There is a broad consensus that in migraine the cortex is hyperexcitable (10) and, historically, photophobia in migraine has been attributed to cortical perturbations (11). However, the relatively recent discovery of intrinsically photosensitive retinal ganglion cells (ipRGCs) has generated a number of studies linking retinal mechanisms to photophobia in migraine. The ipRGCs respond to the ambient light intensity rather than contrast (although some of the five subtypes of ipRGC have also been found to potentially respond to contrast [12]). Therefore, we will discuss both potential retinal and cortical mechanisms of migraine photophobia in turn, and argue that a cortical mechanism explains photophobia from all types of visual stimulation (bight light, flicker, colour, patterns), whereas the retinal mechanisms do not.

## Retinal mechanisms of migraine photophobia

The cones, rods, and the intrinsically-photosensitive retinal ganglion cells (ipRGCs) have all been implicated in photophobia, see a review by Noseda et al (13). We begin by considering the ipRGCs.

One of the original arguments for a retinal mechanism for photophobia in migraine arose from a report of an individual who did not have migraine but who was blind and nevertheless experienced photophobia – she could not perceive light due to the removal of a pituitary adenoma but reported discomfort when light was shone into the eyes. This case was taken as evidence for surviving ipRGCs which do not contribute to conscious visual perception (14). Support for non-image forming ipRGCs remaining active in the blind comes from a case study reporting two blind patients with functionally inactive rods and cones in whom short-wavelength light was able to reset the circadian rhythms. In one of the patients, short-wavelength light increased alertness. The other patient could reliably tell when short-wavelength light was being shown to her and her pupils responded (15). Consequently, Noseda and colleagues (16) investigated photophobia in blind individuals with migraine. They identified 20 such individuals and found that 14 could perceive light despite not being able to see images. All 14 experienced photophobia during their migraine with six experiencing discomfort (four individuals) or ocular pain (two individuals) in between migraine attacks. Cases such as these led to the hypothesis that the response to light of the ipRGCs might be the source of photophobia in general and more specifically in migraine (1).

The ipRGCs subserve entrainment of circadian rhythms (17), affect mood (18), and provide the afferent input for the pupillary light response (19). Although the pupil light reflex has been found to be abnormal in migraine, the findings have been linked to dysfunction of the autonomic nervous system (20). Increased ipRGC activation due to light stimulation has been linked to behavioural aversion in mice (21–23), although mice are nocturnal animals and the aversion may not be a valid model for photophobia in man. In a recent haemodynamic study of individuals with migraine, the spectral composition of ambient light was modulated using silent substitution to selectively excite ipRGCs while keeping constant the activation of cones responsive to short (S), medium (M), and long (L) wavelengths (the metamerism method). The haemodynamic response in the visual cortex was measured using near infrared spectroscopy. When an artificial pupil was used, the haemodynamic response to ipRGC-activating light was large compared to non-ipRGC-activating light, and selectively so in patients with migraine (24). ipRGCs contain the light sensitive opsin melanopsin which is sensitive to shorter wavelengths than rod and L and M cone opsins, being maximal at about 480nm (25). However, it is important to note that the dominant input to the ipRGCs is from the rod and cone photoreceptors (26,27). The time course of intrinsic activation differs from that of the photoreceptors (28) and the ipRGCs may have a role in modulating the output of photoreceptors through amacrine cell activity (29). It remains uncertain how the intrinsic activation of ipRGCs could generate a cortical response different from that from rod/cone activation.

Individuals with migraine have been shown to exhibit increased sensitivity to white, blue, amber or red light, but less to green light, at least during the headache phase, possibly implicating the cone photoreceptors (27). The lack of specific sensitivity to blue light and improvement with green light (compared to red, for example) seems to suggest that direct photoactivation of melanopsin in ipRGCs may not be solely responsible for photophobia in migraine. When measured using a simultaneous recording of the electro-retinogram (ERG) and cortical visually evoked potentials (VEP) in migraineurs, and multi-neuron recordings of the thalamus in rats green light has been shown to evoke the smallest response in cones, in the thalamus and in the visual cortex compared to light of other colours (27). As discussed subsequently (30,31), for the recordings in migraineurs, pupil diameters were not measured and background colours were not specified; it is possible that pupil size, and therefore retinal illuminance, varied between the different colours of stimuli, though they were matched for photopic luminance at the cornea. Also, drawing conclusions regarding human thalamic responses from rodent recordings is challenging due to differing spectral sensitivities.

Rod-driven pathways have also been implicated in photophobia. Bernstein et al. (32) found that both light- and dark-adapted b-wave amplitudes were larger in migraineurs compared with healthy control participants. Whilst the dark-adapted b-wave derives from signals in rod-driven ON bipolar cells, the light-adapted b-wave derives from cone-driven bipolar cells (assuming rods are in saturation). The cone-driven 30 Hz flicker responses did not differ in amplitude, although visual inspection of the traces suggests a possible difference in peak time. Abnormalities in migraine of the amplitude and latency of VEP components to both pattern (33) and flash (34) were first reported more than 40 years ago and have been confirmed in numerous subsequent studies. Although there are undoubtedly some inconsistencies in the findings, which may depend upon such factors as whether migraine is with or without aura, and the time interval since the last attack, the general conclusion that VEPs are abnormal has largely been confirmed. The normal VEP results in the study by Bernstein et al. (32) were therefore exceptional. Also unusual in this study was the finding that some of the individuals with migraine did not show a P2 in the VEP – the 25^th^ percentile being close to zero in their Figure 4. In general, a rod-based mechanism could not sustain photophobia under photopic conditions, where the rods are presumably silent (35). We suggest that mechanisms of photophobia based exclusively on either rod or cone function cannot explain how blind migraineurs experience photophobia if their rods and cones are destroyed (16) unless the activity of ipRGCs is well integrated with that of rods and cones. There is evidence this is indeed the case (26,36). Noseda et al. (13) have recently proposed that photophobia can arise from any class of photoreceptor, which suggests that the basis for photophobia arises not just from the ipRGCs but may lie elsewhere, possibly in the visual cortex, as we will discuss later.

The idea of a retinal basis for photophobia has been attractive partly because there is an indirect pathway between the optic nerve and the trigeminal nerve (particularly in the case of the ipRGCs [37]) and subcortical structures such as the basal ganglia, the thalamus, and the hypothalamus (16,38) proposed in a review (38). Note, that while these studies do not focus on migraine, the mapping of the pathway generates a potential mechanism linking photophobia to pain in migraine. This direct subcortical connection has been used to explain some of the effects of photophobia on appetite and on mood that are associated with migraine (38). Indeed, the trigeminal nerve has been implicated in migraine pain more generally (39,40).

It is possible, even likely, that there are different forms of photophobia that have different mechanisms, with migraine photophobia differing from that in ocular disorders (1), given the wide range of visual stimuli apart from bright light to which individuals with migraine are susceptible. But even in mouse models of photophobia in ocular disorders, there is some discrepancy as to whether retinal mechanisms are the sole cause of photophobia. Matynia and colleagues (41) in studies of light aversion induced by corneal damage in mice have shown that the behavioural response depends upon the presence of ipRGCs although the effect of opiates in enhancing aversion is independent of ipRGC activity and is more likely to be influencing a central mechanism (42).

Where the irradiance (ambient light level) is the sole or major component in the provocation of light aversion, then the ipRGC system is likely to play a major role, because this is the only system in the retina that can signal irradiance directly. However, this role is likely to be subserved not only by the melanopsin-mediated intrinsic activity of the ipRGCs but also the input to ipRGCs from rod and cone photoreceptors in scotopic and photopic conditions respectively.

In summary, there is evidence of abnormal retinal responses to light in migraine, but there are inconsistencies as to which cells in the retina are implicated and whether abnormal retinal functioning is the sole mechanism for the photophobia. We will now discuss the cortical mechanisms that are associated with migraine photophobia, with particular emphasis on the evidence for aversion, discomfort and headache evoked by flickering light, colour, and spatial patterns. We argue that these types of photophobia are best explained by cortical mechanisms.

## Cortical mechanisms of migraine photophobia

One difficulty with the studies cited above in proposing retinal mechanisms for migraine photophobia is the assumption that photophobia is aversion to light alone. In migraine there is also aversion to, and pain from, flicker, pattern and colour. We will consider the evidence for each of these in turn and argue that the aversion and pain can only be explained by implicating cortical mechanisms.

**Aversion to Flicker:** Aversion to flicker is most pronounced at frequencies at which the flicker is most visible at low contrast and at which it is most epileptogenic (10-20Hz) (43). In general, visual stimulation that is epileptogenic is also migrainogenic (5), although even when flicker is so rapid as to be imperceptible it is known to cause headaches (3). There are many possible mechanisms. One possibility is indirect interference with the control of eye movements due to the spatial pattern formed on the retina during a saccade when the contours in a scene are lit intermittently (44). This intra-saccadic pattern is visible with flicker at frequencies as high as 11 kHz, particularly in individuals who have visual discomfort (45). Perception during a saccade is used by the brain to guide eye movements (46), and the intra-saccadic spatial pattern from flicker may interfere with this mechanism.

**Aversion to Patterns:** Even under steady lighting, patterns of stripes can have aversive properties. Black and white stripes of a particular size and spacing are generally uncomfortable, and particularly so for individuals with migraine (4,5). The patterns evoke illusions that are related to headaches both in terms of frequency (the higher the frequency of headaches, the greater the number of illusions) and any lateralisation of the pain (when the pain is lateralised the illusions predominate in one homonymous visual hemifield)(5). The patterns responsible for headaches are very similar to those that trigger seizures (5). For example, the spatial frequency (stripe spacing) at which aversion is maximal is about 3 cycles per degree (cpd) irrespective of viewing distance (47). Haemodynamic responses to mid-range spatial frequencies are larger than to other spatial frequencies in normal subjects and this effect is exaggerated in migraine (48,49); (the relatively low spatial frequency at which Huang et al obtained a maximal BOLD response is attributable to the low mean luminance employed.) The pattern ERG (which reflects retinal ganglion cell function) has maximal amplitude at a spatial frequency of about 1.5 cpd (50) somewhat lower than that at which discomfort is maximal (5), although, interestingly, one study reported altered pattern ERG parameters (smaller P50, and smaller, more delayed, N95 components) in migraine (51).

Most of the above observations are consistent with other convergent evidence for cortical hyper-excitability in migraine (10,52). Indeed the illusions seen in epileptogenic patterns may provide a simple clinical correlate of the hyper-excitability - they predict the susceptibility to out-of-body experiences in the general population, for example (53). Pattern-related photophobia may be affected by any visual deficits in contrast sensitivity that sometimes occur in migraine (54) and the change in sensitivity to peripheral targets that can follow an attack (55). Nevertheless, performance of some tasks such as the discrimination of grating contrast can be supra-normal interictally (7), consistent with hyper-excitability.

**Aversion to Colour:** Coloured stripes are generally aversive (56) and again, particularly so for individuals with migraine (6). The aversion increases with the difference in colour between the stripes (colour contrast), even when the stripes have the same luminance (56). The larger the difference in colour the greater the amplitude of the haemodynamic (56) and electrophysiological (57) responses the patterns evoke in normal subjects. The increase in discomfort and evoked potential amplitude is greater in individuals with migraine than in controls (8). The simple relationship between discomfort, amplitude and colour difference occurs only when the colour difference is expressed in terms of the *Commission Internationale de l’Eclairage* (CIE) uniform chromaticity scale (UCS) diagram, and not when the difference in colour is expressed in terms of cone contrast (57). In other words the effect of colour differences on discomfort depends upon the post-processing of colour in the visual pathway (58) rather than the amplitude of the photoreceptor response. Maps that resemble the UCS chromaticity diagram have been identified in Visual Area 2 (V2) of the visual cortex in the monkey (59). The relationship between discomfort and colour difference is therefore consistent with a cortical rather than a retinal mechanism.

The sensitivity to flicker, patterns and colour can be interpreted as reflecting the cortical hyper-excitability with which migraine is associated. All three sources of stimulation have been shown to evoke a cortical response, and one that is large in migraine. Nevertheless, photophobia is typically thought of as a sensitivity to bright light. The work of Bargary and others (60) suggests that this “traditional” concept of photophobia may also be attributed to cortical hyper-excitability. The discomfort glare threshold in response to peripheral lights was measured and used to divide observers into those who were sensitive and those who were less so. The sensitive group exhibited a larger BOLD response in the cunei, the lingual gyri and the superior parietal lobules. The authors argued that the discomfort glare that was being measured might be a reflection of a hyper-excitability or saturation of visual neurons.

**Figure 1.**
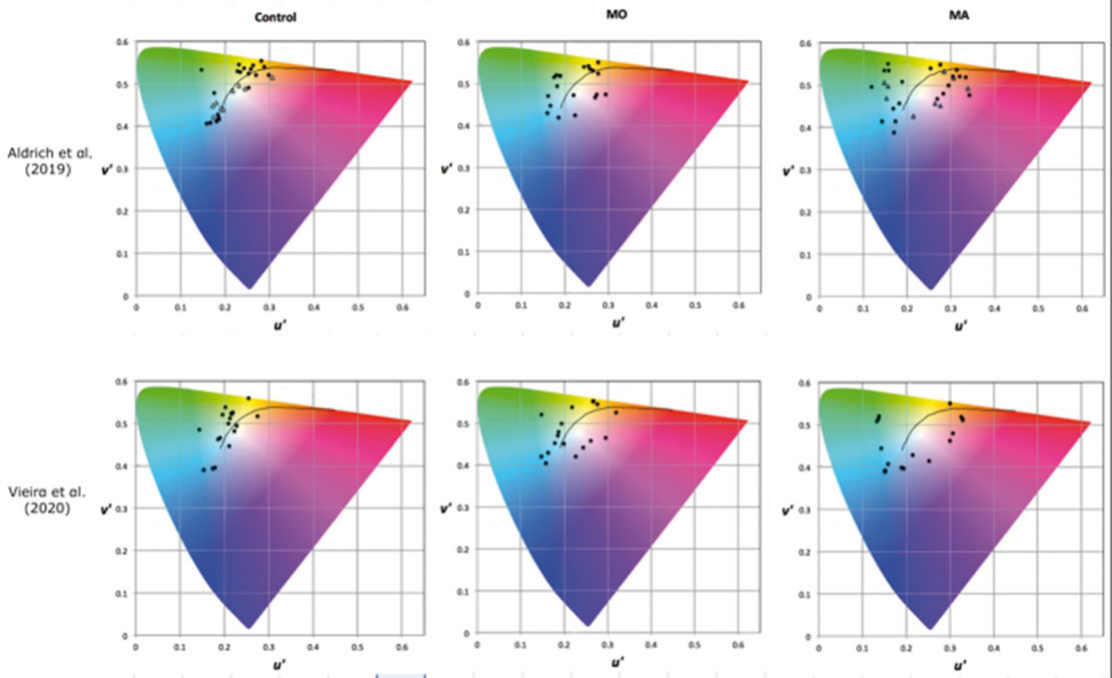
Data from Aldrich et al. (7) (top row) and Vieira et al. (9) (bottom row). Each point shows the chromaticity of light chosen as comfortable for reading by individuals without migraine (Column 1), individuals who experienced migraine without aura (Column 2) and individuals who experienced migraine with aura (Column 3). All assessments were interictal. The continuous line shows the daylight locus.

Another aspect of the influence of colour is that the aversion to patterns can be reduced by coloured lighting although the optimal chromaticity varies from one observer to another (61,62). In healthy observers and those who experience migraine without aura the chromaticity chosen almost invariably lies close to the daylight locus, see Figure 1 (left column), although some individuals choose a yellowish light and others a blue. In patients who experience migraine with aura, however, the chosen chromaticity usually lies well away from the daylight locus and has a strong saturation (7,9), *see* Figure 1 (right column). The distribution of the chosen colours is not related to the energy captured by the ipRGCs (9). The chosen colour normalises the otherwise abnormally low contrast discrimination thresholds in patients with migraine (7) and improves visual search (9). It also normalises the otherwise abnormally large haemodynamic response (49), possibly because of the manner in which colour is represented cortically (58,59,63). If photophobia is indeed a manifestation of cortical hyper-excitability then there is no reason to suppose that the hyper-excitability is uniform throughout the cortex. In patients with pattern-sensitive epilepsy, for example, the seizure trigger appears to involve complex cells with a limited range of orientations (64), suggesting that the hyper-excitability can involve subsets of visual neurons differentially. The limited knowledge we have of cortical processing of colour suggests that in visual areas such as V2 the cells are arranged as per a perceptual map of colour rather similar to the CIE UCS diagram (58,59), so it is quite possible that changing the chromaticity of the illuminating light alters the distribution of activity within the visual cortex. We hypothesise that when the chromaticity is regarded as “comfortable”, the distribution avoids local areas of hyper-excitability. Early observations suggested that it is the chromaticity of light (its unchanging physical properties) rather than its subjective colour appearance that determines the clinical benefit of coloured filters (65). Colour appearance takes account of the illumination to provide for colour constancy, and this processing occurs in more anterior visual areas such as V4 (66). The clinical effect of the filters may therefore depend on activity in earlier posterior visual areas of the cortex, such as V2 (58). The effect of such filters would be to reduce the average chromaticity difference between contours in the retinal image, and this is known to reduce discomfort quite generally (67) as well as in migraine(49).

## Cortical mechanisms of photophobia are parsimonious

It is becoming clear why glare, flicker, patterns, and colour have these unfortunate effects. The human visual system evolved to process scenes from nature. Natural images have a particular statistical structure (68) that the visual system processes efficiently. It uses a sparse code such that few neurons fire at any given time, conserving metabolic energy (69). Computational models of the visual system suggest that striped patterns reduce the sparseness, increasing “neural” activity (70). When images have an unnatural statistical structure they are aversive (71–74) and patterns of stripes are perhaps the least natural of all visual stimuli. Measurements of images have been undertaken in terms of the Fourier amplitude spectrum (73), the orientation spectrum (75) and chromaticity difference (67) and images with statistics outside the range typical of natural images have been associated with discomfort. Photophobia can therefore be seen as an exaggeration of this sensory discomfort, at least interictally. The photophobia that occurs during a migraine attack may well have a wider variety of mechanisms and is more difficult to study.

Attempts to separate the stimulation of the ipRGCs from the stimulation of other photoreceptors by use of unusual spectral power distributions (76) involve atypical covariance in the response of the various photoreceptors and downstream neurons. As we have seen, un-natural stimulation is often uncomfortable, particularly so for individuals with migraine, and this may detract from inferences regarding the role of the ipRGCs in migraine.

Light-induced damage to the retina is a well-established concept and light avoidance behaviour must in part be related to prevention of retinal damage (77). The mechanisms of pain in this context may well differ from those proposed here as explanations of migraine photophobia. Nevertheless, visual stimuli that give discomfort, pain or seizures are strong stimuli in the sense that they evoke a large cortical haemodynamic response in normal observers (5,48,74). Teleologically, discomfort and pain usually signal potential damage to the organism. It has been argued that visual discomfort is no different and may be a homeostatic response to reduce damaging hypermetabolism (78). If so, then photophobia in response to bright light, flicker and patterns can all be seen as a homeostatic response which is on a continuum of severity in the population. According to this view individuals who exhibit photophobia have a high rate of metabolism (consistent with other evidence of cortical hyper-excitability) that is then further exacerbated by visual stimulation. The larger BOLD response in individuals who experience discomfort glare (60) and in patients with migraine (79–81) or visual stress (82) is consistent with such a viewpoint. It is currently accepted that small cerebral vessels and pia mater are insensitive to pain in humans and that intracranial pain-sensitive structures are limited to the dura mater and its feeding vessels, large venous sinuses and proximal parts of the large arteries of the circle of Willis (40,83). This view has recently been challenged by prospective collection of intra-operative reports of pain, demonstrating that small cerebral vessels and/or sulcal pia mater are sensitive to mechanical stimulation. The pain is mostly referred in the V1 territory of the trigeminal nerve (84). It is a small step to propose that the enlarged haemodynamic response to aversive stimuli observed in individuals with migraine provokes pain by distension of small cerebral vessels. To quote the recent study: “The sensory nerve fibres around cranial vessels contain to a varying degree calcitonin gene-related peptide (CGRP), substance P, neurokin A and are likely to play an important role in head pain of a migraine attack.” (84).

## Closing remarks

The above review has considered photophobia in migraine only and has brought together the various components of visual discomfort that occur, under the assumption that cortical hyper-excitability provides a parsimonious common mechanism, at least for the interictal photophobia. The photophobia that occurs during a migraine attack is more extreme and may involve extra-cortical mechanisms. A limitation of the studies we have cited is that they have usually collected interictal data over relatively short time periods. Their findings may not reflect the performance of the visual system following hours in the dark, when longer term adaptive processes may ensue. Moreover, photophobia is a symptom in many disorders and cortical hyper-excitability is unlikely to provide a general explanation. Perhaps comparisons of the electroretinal and electroencephalographic response to light and pattern in the wide variety of conditions in which photophobia occurs will help to elucidate the retinal and cortical contributions to these complex symptoms and help identify the mechanisms specific to each condition.

## Article Highlights

- Photophobia in migraine includes sensitivity to spatial patterns, colour and flicker.
- Photophobia can be interpreted as reflecting the cortical hyperexcitability with which migraine is associated.
